# Advances in surface modification of tantalum and porous tantalum for rapid osseointegration: A thematic review

**DOI:** 10.3389/fbioe.2022.983695

**Published:** 2022-09-13

**Authors:** Xi Wang, Wentao Liu, Xinding Yu, Biyao Wang, Yan Xu, Xu Yan, Xinwen Zhang

**Affiliations:** ^1^ Department of Emergency and Oral Medicine, School and Hospital of Stomatology, China Medical University, Liaoning Provincial Key Laboratory of Oral Diseases, Shenyang, China; ^2^ Shenyang National Laboratory for Materials Science, Institute of Metal Research, Chinese Academy of Sciences, Shenyang, China; ^3^ The VIP Department, School and Hospital of Stomatology, China Medical University, Liaoning Provincial Key Laboratory of Oral Diseases, Shenyang, China; ^4^ The Comprehensive Department of Shenyang Stomatological Hospital, Shenyang, China; ^5^ Center of Implant Dentistry, School and Hospital of Stomatology, China Medical University, Liaoning Provincial Key Laboratory of Oral Diseases, Shenyang, China

**Keywords:** tantalum, porous tantalum, surface modification, surface nanostallization, surface coating

## Abstract

After bone defects reach a certain size, the body can no longer repair them. Tantalum, including its porous form, has attracted increasing attention due to good bioactivity, biocompatibility, and biomechanical properties. After a metal material is implanted into the body as a medical intervention, a series of interactions occurs between the material’s surface and the microenvironment. The interaction between cells and the surface of the implant mainly depends on the surface morphology and chemical composition of the implant’s surface. In this context, appropriate modification of the surface of tantalum can guide the biological behavior of cells, promote the potential of materials, and facilitate bone integration. Substantial progress has been made in tantalum surface modification technologies, especially nano-modification technology. This paper systematically reviews the progress in research on tantalum surface modification for the first time, including physicochemical properties, biological performance, and surface modification technologies of tantalum and porous tantalum.

## Graphical Abstract

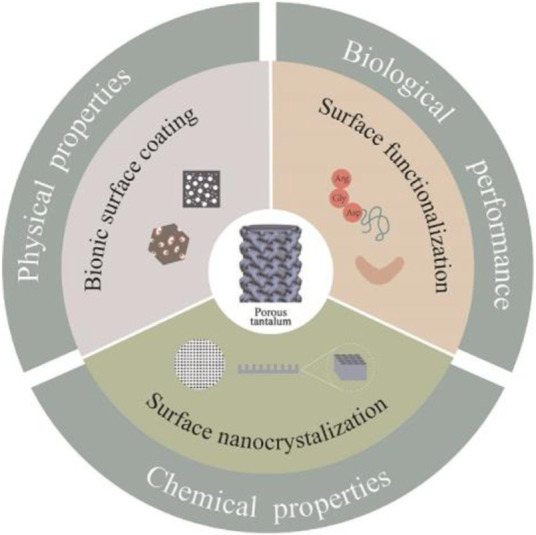

## 1 Introduction

Bone defects are often caused by trauma, infection, and tumors, among others (Locker et al., 2018). After bone defects reach a certain size, the body can no longer repair them; these are referred to as critical bone defects, the restoration of which requires autogenous bone transplantation, allogeneic bone transplantation, or synthetic grafts (Ho-Shui-Ling et al., 2018). Autologous bone is regarded as a “gold standard” for the rebuilding of bone defects, but source and donor complications restrict its application. Rejection, disease transmission, and high cost are among the limitations of allografts (Qian et al., 2020). Meanwhile, the application of artificial synthetic bone is limited by poor mechanical properties (Tang D. et al., 2016). At present, metal is particularly valuable for implantation of bone defects because of its advantages including the source of the material is not restricted, good mechanical properties, and good biocompatibility. Currently, tantalum and titanium alloy, cobalt-chromium alloy, and stainless steel are metal materials most widely used for medical applications. However, titanium was found to have weak effects on osteoinduction and osseointegration (Lu et al., 2018; Guillem-Marti et al., 2019; Souza et al., 2019; Xue et al., 2020). Meanwhile, cobalt-chromium alloy has an elastic modulus of 220 GPa, which greatly limits its use. There is only limited interaction between stainless steel and host bone, and no bone tissue attachment or bone bonding occurs on the surface (Lu et al., 2018). Against this background, tantalum has received increasing attention for its better corrosion resistance, protein adsorption and hydrophilicity, and ability to induce bone formation compared with titanium (Lu et al., 2018; Wang et al., 2021). Porous tantalum is also a promising biomaterial with a structure similar to cancellous bone in the human body (Levine et al., 2006). Tantalum has become another new biological material after titanium, which is widely used in pacemaker electrodes, artificial spines, dental implants, artificial joints, radioactive marking, nerve repair nets, heart stents, hemostatic materials, and other medical applications (Park et al., 2020).

Good implant materials should have excellent effects of promoting osteogenesis, osteoinduction, osteoconduction, and osseointegration. Osseointegration involves the direct integration of bone and material, in which structural and functional integration between the living bone and the implant surface occurs (Overmann et al., 2020). After medical metal materials are implanted into the body, a series of interactions occurs between the material’s surface and the microenvironment. The nature of this interaction is mainly determined by the surface topographic characteristics and the chemical composition of the implant surface (An et al., 2019). Given that tantalum is a bioinert metal, to achieve faster and earlier bone in-growth and more stable bone integration (Li and Wang, 2020), especially for patients with systemic diseases, many methods have been proposed to modify the implant surface. For tantalum and titanium implants, such modification methods include sandblasting, anodic oxidation, and alkali heat treatment. Appropriate surface modification can also improve the corrosion resistance, mechanical properties, and biological properties of implant materials (Liu et al., 2004; Zhang L.-C. et al., 2020; Xu C. et al., 2021). Different modification methods provide multiple directions for the study of tantalum interface effect (Li et al., 2009). Substantial progress has been made in tantalum surface modification, especially nanometer modification, but to the best of our knowledge, no summary of tantalum surface modification methods has been published. This paper systematically reviews the progress made in research on tantalum surface modification, to broaden researchers’ understanding of tantalum value-added design, and to promote the wider clinical application of tantalum as a potential bone substitute. The main forms of tantalum surface modification technology include surface nanocrystallization, surface functionalization involving bioactive ingredients, and bionic surface coating. This review introduces tantalum, including its porous form, in terms of the physicochemical properties and biological performance, and progress in research on surface modification technology.

## 2 Physicochemical properties and biological performance

Tantalum was first discovered in 1802 by Anders Gustav Ekeberg, a Swedish chemist. He named this new element after the Greek mythological figure Tantalus. Werner first purified tantalum in 1903 and obtained relatively pure samples (Weeks, 1932). Tantalum is an unusual transition element (atomic number 73, molecular weight 180.05). The density of solid tantalum is 16.68 g/cm^3.^ At ambient temperature, tantalum is a shiny blue-gray and malleable metal with rigidity of 6–6.5 (Maccauro et al., 2009). As a refractory material, tantalum has a fusing point of up to 3,017°C (Sopata et al., 2019). Tantalum has been widely used in aircraft, rockets, and for other purposes requiring heat-resistant materials, as well as in industrial fields requiring high-strength parts, given its excellent thermal and electrical conductivities (Balla et al., 2010). Tantalum is easily oxidized in air and forms a chemically stable, dense Ta_2_O_5_ passivation layer on its surface, usually with a thickness of 2–3 nm. This layer prevents metal ion leaching and reduces partial inflammation arising from corrosion products, making the tantalum surface resistant to erosion (Zhang L. et al., 2020). Indeed, tantalum is well resistant to chemical corrosion by most strong acids, including hydrochloric and nitric acids, at temperatures not higher than 150°C. Only hydrofluoric acid, fuming sulfuric acid, and potassium hydroxide are known to cause significant corrosion to tantalum metal (Fattah-Alhosseini et al., 2017). The naturally occurring surface oxide layer of Ta_2_O_5_ also confers excellent biological properties on tantalum. Despite an absence of intrinsic antibacterial properties (Harrison et al., 2017), compared with stainless steel and titanium, tantalum is less colonized and adhered to by bacteria because of its natural oxidation surface layer (Ta_2_O_5_) (Shimabukuro et al., 2019). Numerous studies have illustrated that hydrophilic surfaces promote cell proliferation and adhesion more (Boyan et al., 2017). The formation of Ta_2_O_5_ changes the hydrophobicity of tantalum, which is closely connected with the crystal form of Ta_2_O_5_ (Huang et al., 2014).

The elastic modulus of natural cortical bone (12–18 GPa) and cancellous bone (0.1–0.5 GPa) in the human body are much lower than the elastic modulus of solid tantalum (186 GPa). Huge differences in elastic modulus may lead to stress-shielding effects, which involve bone loss around implants with a high elastic modulus. This eventually causes the implant to become loose and detach (Li J. et al., 2020). The emergence of porous tantalum materials has resolved these problems. As early as the 1990s, Kaplan developed tantalum implant material with a porous structure (Kaplan Richard, 1993). Then, in 2003, Zimmer company attached pure tantalum powder to a pre-prepared vitreous carbon skeleton by the chemical vapor deposition (CVD) process, and then removed the carbon skeleton to obtain an innovative tantalum medical material, which was named trabecular metal and applied to the field of bone surgery (Ling et al., 2018). Soon after, many manufacturers also engaged in producing porous tantalum, such as Printing Additive Manufacturing Co., Ltd. (Zhuzhou, China) (Wang et al., 2019).

Porous structures similar to natural bone offer space for cell activity, as well as for nutrient exchange, bone-inducible proteins, and blood vessel formation, and for inward bone growth and bone integration (Cheng et al., 2021). The elastic modulus of porous tantalum (2.4–3.9 GPa) is between that of human cortical bone (12–18 GPa) and cancellous bone (0.1–0.5 GPa). Compared with that of titanium alloy (106–114 GPa), the elastic modulus of porous tantalum can effectively reduce the shielding effect, which is more conducive to maintaining bone density around implants and reducing long-term bone loss around implants (Hosoki et al., 2018). Porous tantalum has adjustable mechanical properties. Its elastic modulus and strength can be reduced by increasing its porosity, which means that porous tantalum can be created with various voids, especially for plastic surgery implants. The pore size of porous scaffolds also affects the behavior of cells and the growth of bone. The optimum pore size also differs depending on the cell. Some researchers have asserted that cell adhesion decreased with increasing pore size and that the highest levels of cell attachment were found on the scaffolds with the smallest pore size (96 µm) (Murphy and O’Brien, 2010). Elsewhere it was suggested that pores greater than 300 µm are essential for bone in-growth (Roosa et al., 2010). Making a balance between mechanical and biological properties of porous tantalum by regulating a reasonable pore/pore ratio is a key difficulty for future research and development (Huang et al., 2021). The maximum bending strength of porous tantalum is 110 MPa, which can provide sufficient physiological support for newborn bone tissue. The friction coefficient between cancellous bone and porous tantalum is 0.88, and that of cortical bone is 0.47, which is 40–80% higher than those of other conventional metals, initially making porous tantalum implanted into host bone more stable (Bencharit et al., 2014). By optimizing the structure and fabrication process of tantalum, the limitations associated with the mechanical and physical properties of tantalum can be overcome, increasing tantalum’s clinical application potential.

Biocompatibility is defined as when a material does not cause declines of cell and tissue function, and does not cause inflammation, carcinogenesis, or a rejection reaction after contact with living tissue and body fluid. Tantalum was introduced for medical use as early as 1940 (Burke, 1940). Tantalum-coated implants were immersed in phosphate-buffered saline for 28 days and the findings showed that they released only 0.2 μg/L tantalum (Zhu et al., 2017). Macrophages, as components of the innate immune system, are the first immune cells that participate in sterile loosening. Some researchers indicated that tantalum particles were absorbed through phagocytosis *in vitro*. Macrophage activity increased gradually on days 1 and 3 in the presence of 20 μg/ml tantalum particles. The study also showed that tantalum particles maintained high cell viability even at higher concentrations (50–500 μg/ml) and longer incubation times (up to 7 days) (Zhang L. et al., 2020). Similar results (Wang P. et al., 2020) were presented. Moreover, Kang et al. (Kang et al., 2017) found that low concentrations (<20 μg/ml) of tantalum particles promoted the proliferation of MC3T3-E1 osteoblasts in mice by inducing autophagy. Soon thereafter, this team suggested that the Akt/mTOR signaling pathway and its feedback loop are related to nanotantalum-induced autophagy and proliferation of MC3T3-E1 cells (Kang et al., 2021).

Bone marrow mesenchymal stem cells (BMSCs) (Wei et al., 2016; Lu M. et al., 2019), MG63 osteoblasts (Wang et al., 2015; Bakri et al., 2019), and dental pulp stem cells (Bakri et al., 2019) were each co-cultured with porous tantalum to evaluate its biological activity, which was shown to result in better cell morphology, adhesion, proliferation, and osteogenic differentiation. The long-term survival of metal implants *in vivo* depends not only on the good integration of bone and bone ingrowth, but also on the adjacent soft-tissue structure. Some researchers (Zhao et al., 2021) fabricated an integrated three-dimensional scaffold material utilizing porous tantalum (pTa) and nano-gelatin (GNP) hydrogel, and inoculated endothelial cells (ECs) derived from BMSCs for vascular tissue engineering. A stable capillary-like network was observed 4 weeks after implantation in nude mice. The pTa-GNP hydrogel scaffolds were found to be biocompatible with the host and possessed biomechanical characteristics and angiogenicity. In addition, tantalum has been found to exert no harmful effects on L929 mammalian cells (Wauthle et al., 2015), human monocytic leukemia cells (THP-1) (Yang et al., 2021), gingival fibroblasts (Zhang C.-n. et al., 2021), and fibroblasts (Tran et al., 2017; Uslu et al., 2020), and enhanced the integration of soft tissues around implants. For a recent animal experiment, some researchers (Lei et al., 2022) fabricated a new type of porous tantalum scaffold with high interfacial strength directly on solid Ti6Al4V substrate. This scaffold exhibited good biomechanical properties and the interface bonding strength reached 447.5 MPa When the scaffold was implanted into a rabbit femur defect, imaging and histological examination confirmed abundant new bone formation and bone growth. Similarly, good biocompatibility and osteogenic properties of porous tantalum have been observed in goat and rabbit models (Wang et al., 2015; Wei et al., 2019). Tantalum is currently considered to have satisfactory biocompatibility (Loechel et al., 2019; Bilandzic et al., 2020; Park et al., 2020).

It is well known that, after a metal material for medical purposes is implanted into the body, a series of interactions occurs between the microenvironment and the material’s surface. The interaction between cells and the implant surface mainly depends on the topographic characteristics and chemical composition of the implant surface (An et al., 2019). Studies have shown that materials with a rough surface, high hydrophilicity, and high surface energy are more able to promote bone formation and bone bonding (Shah et al., 2019; Hu et al., 2021). For successful orthopedic implantation, especially under conditions with poor or inadequate bone, it is preferable to modify and design the surface morphology of implant material in order to regulate and influence cell behavior, especially for improving rapid osteogenicity (Li and Wang, 2020). For example, classical electrical discharge machining (EDM) can not only improve the corrosion resistance and fatigue resistance of cobalt-chromium implant, Ti-6Al-4V and β-Ti alloys, but also deposit biocompatible film on a metal substrate, so as to promote bone in-growth, cell proliferation, and improve biocompatibility (Bhui et al., 2018; Mahajan and Sidhu, 2019; Sidhu et al., 2021). Research on the surfaces of metal materials for medical purposes is also currently a hot topic in the fields of biomaterials and biomedical engineering. A large number of researchers have attempted to improve the surface properties of tantalum through different surface treatments. A review of the literature published in the last 10 years has shown that the main technologies for modifying the surface of tantalum include surface nanocrystallization, surface functionalization involving bioactive ingredients, and bionic coating.

## 3 Surface nanocrystallization

Researchers have analyzed the multilevel arrangement of natural bone tissue in the human body through nanobiotic characterization. Bone tissue is typically composed of three structures at the molecular level: 1) nanostructures (from a few nanometers to hundreds of nanometers) consisting of non-collagen organic protein, fibrous collagen, and embedded mineralized crystals (hydroxyapatite); 2) microstructures (1–500 μm), including lamellar bone, bone unit, and Harvard system; and 3) macrostructures, including cancellous bone and cortical bone (Ye et al., 2020). With the success of bionics in various fields. In addition to conventional methods such as sandblasting and acid etching to roughen the implant surface (Mahajan and Sidhu, 2018; He et al., 2019; Xu A.-t. et al., 2021), an increasing number of researchers have simulated and constructed structures similar to natural bone on the implant surface from the perspective of bionics (Saita et al., 2016).

Metallic nanostructured materials are single-phase or multiphase metallic materials whose basic structural characteristics are on the nanometer scale (<100 nm), such as nanocrystalline material (3D grain size on the nanometer scale) Nanotwin material (twinning - matrix lamellar thickness in nanometer scale), nano-lamellar structure (2D layer structure with a thickness on the nanometer scale), gradient nanostructures (grain size from nano-scale gradient changes to macro-scale), and hybrid nanostructures (nano-grain mixed with coarse grain structure). According to the physical and chemical structures and phase structure characteristics, nano-biological materials can be divided into nanocrystals, nanoparticles, and nanocoatings. When the size of a material reaches the nanometer level, it exhibits special effects differing from those at the macroscopic size, which is referred to as the nanometer size effect.

In the last 10 years, research on the interaction of nanopores (<100 nm) with cells has generated considerable interest. Pore sizes span a range of biologically important dimensions, such as those of small DNA fragments as small as tens of nanometers, and important extracellular matrix (ECM) proteins such as fibronectin and albumin, which are often in the 100 nm range. Different pore sizes have different effects on cells. Thus, the pore size can be adjusted to suit the apertures required by certain cell behaviors (Lutolf et al., 2009). Nanotechnology can also improve many properties of metal materials used for medical purposes. Nanocrystals have strong hydrophilic and protein-adsorbing properties, excellent resistance to electrochemical corrosion and passivation behavior, and can improve the service life of metal implants. These features have made nanomaterials an important hotspot and focus of development in materials science, especially biomaterials (Souza et al., 2019).

The methods of preparing nanostructures of bulk materials can generally be divided into two categories: “bottom-up” and “top-down” methods. The “bottom-up” approach involves converting the initial target into atoms, molecules, or ions by a physical or chemical method, and then transferring them to the area of deposition under the action of an external field to form nanostructures. This method has been widely used in the preparation of thin film nanomaterials, but there are still great difficulties in the preparation of large bulk materials. Meanwhile, the “top-down” approach is also known as plastic deformation technology because the conventional coarse-grained structure is broken down to the nanoscale through plastic deformation. This method can be used to prepare large bulk nanostructured materials with high density and no porosity, and it has wide applicability and can realize nanostructures in almost all metal materials (Meyers et al., 2006). The section below introduces several classical tantalum surface nanocrystallization technologies.

### 3.1 Plasma immersion ion implantation

**FIGURE 1:**
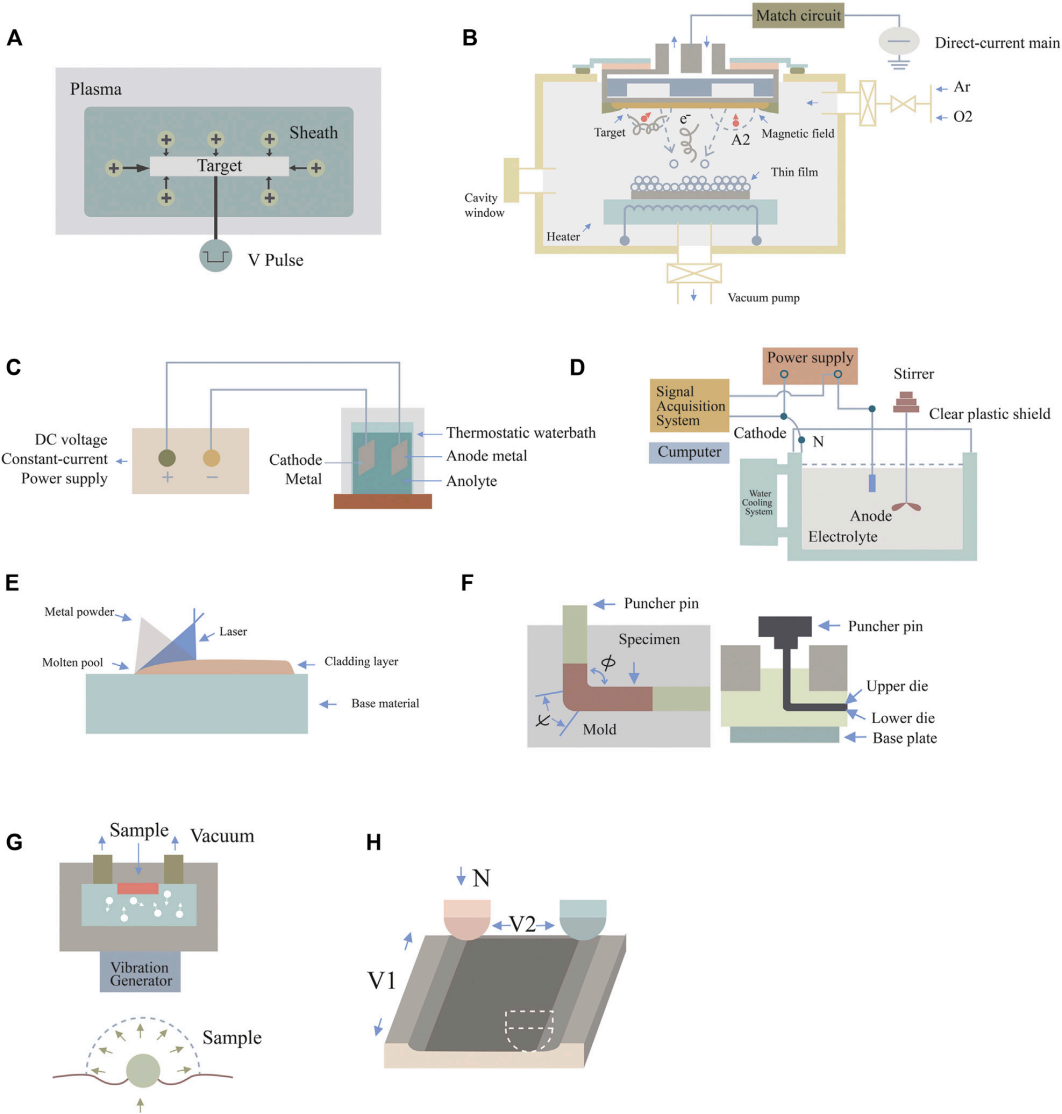
Schematic diagram of surface nanotechnologies. **(A)** Plasma immersion ion implantation, **(B)** DC magnetron sputtering technology, **(C)** anodic oxidation technique, **(D)** micro-arc oxidation technology, **(E)** laser cladding technology, **(F)** equal channel angular pressing, **(G)** surface mechanical attrition treatment, and **(H)** sliding friction treatment.

Plasma immersion ion implantation (PIII) has been widely applied in precision manufacturing, aerospace, and biomedical engineering. It works as shown in Figure 1. The specimen is immersed in plasma, a negative pulse voltage is applied to the target, and electrons around the target are rapidly squeezed out, leaving positive ions to form a critical sheath near the surface of the object. The ions enter the surface in all directions and uniformly under the electrostatic force of the electric field. With the introduction of different elements and functional groups, surface properties such as cytocompatibility, antibacterial activity, and mechanical properties can be selectively adjusted. However, the disadvantage of this technology is that the coating can easily strip the substrate (Jin et al., 2014).

Some researchers (Park et al., 2019a) reported a novel surface-nanoengineered implant, in which nanostructured tantalum (nano/Ta) was introduced onto the surface of silicone by sputtering plasma immersion ion implantation (S-PIII), thereby improving the bioinertia and hydrophobicity of silicon. *In vitro* and *in vivo* experiments demonstrated that nano/Ta silicone rubber implants had good affinity for fibroblasts. By analyzing the number of macrophages, the differentiation and activation of myofibroblasts, and the density of collagen, it was proven that nano/Ta silicone rubber implants can reduce the formation of fibrous capsules and improve biocompatibility. Polyether ether ketone (PEEK) (Wang et al., 2022) is known to have an elastic modulus similar to that of bone, but its bioinertia and osteogenic ability are poor. Lu et al. used PIII to inject tantalum ion energy into PEEK and form Ta_2_O_5_ nanoparticles on the surface (Lu et al., 2015). The surface elastic modulus of PEEK implanted with Ta ions was found to be closer to that of human cortical bone. *In vitro* and *in vivo* experiments also confirmed that Ta-PIII-modified PEEK could promote osteogenic differentiation and enhance the osteogenic ability of PEEK. Therefore, it has great potential for application in dental and orthopedic implants. On this basis, studies have compared the effects of tantalum ions with those of other ions. Wang et al. employed plasma immersion ion implantation and deposition (PIII&D) to inject tantalum and calcium into porous titanium (Wang et al., 2016). Highly consistent results at the molecular, genetic, and protein levels suggested that a more stable and durable osteogenic effect occurred in the tantalum implant group. The combination of tantalum and PIII does not cause any adverse changes in mechanical properties, but can fully realize the potential of tantalum, and this potential is stable and durable.

### 3.2 DC magnetron sputtering technology

DC magnetron sputtering technology refers to the mutual effect between a magnetic field and an electric field to make electrons move in a spiral shape near the target surface, so as to increase the probability of electrons hitting argon gas to produce ions. The ions generated then collide with the target surface under the action of the electric field and subsequently sputter out the target. DC magnetron sputtering coating technology, as an energy-saving and efficient way to obtain thin films, is significant for the preparation of nanoscale thin films, and is widely applied in the surface treatment of parts, as well as conferring special optical, electrical, magnetic, and other functional and physical properties on thin films. The disadvantage of this technology is that the tantalum coating is slightly thin, which reduces the utilization rate of tantalum. However, this technology has the advantages of high film deposition speed, simple equipment, easy operation, low-temperature deposition, dense and uniform film material, and high adhesion between film and substrate. This technique greatly improves the issue of coating stripping for ion implantation (Kelly and Arnell, 2000).

Zhu et al. prepared tantalum films with nanomorphological features on the surface of commercial SLA titanium by DC magnetron sputtering for the first time (Zhu et al., 2017). It was reported that tantalum-modified titanium upregulated the expression of specific receptors (VCL, ITGA5, and ITGB1) and Fn1 (an important ECM adhesion protein) on rat bone marrow mesenchymal stem cells (rBMSCs), and conferred good adhesion and diffusion on these cells, which largely depended on the upregulation of adhesion-related genes and the activation of FAK. The tantalum coating also showed good antibacterial activity against *Streptococcus* mutans and Prinomonas gingivalis, with Prinomonas gingivalis exhibiting greater sensitivity, which might be related to the special nanostructure. The rBMSCs were also more likely to adhere and diffuse on tantalum-modified titanium surfaces even when co-cultured with oral pathogens by an innovative co-culture model. This study identified the antibacterial properties of tantalum coating and the mechanisms related to early cell interactions, and applied an innovative co-culture model. It should contribute to the clinical application of nanotantalum coating in implants in a complex oral environment, laying a foundation for future *in vivo* and clinical efficacy studies. In addition to BMSCs a recent study indicated that gingival fibroblasts can also maintain good proliferation and migration on nanotantalum coating prepared by DC magnetron sputtering technology. The underlying mechanism was attributed to increased expression of integrin β1 and activation of FAK in gingival fibroblasts. This study showed that the nanotantalum coating prepared by this technology can also enhance the integration of soft tissues around implants (Zhang C.-n. et al., 2021).

Silver nanoparticles are known to have good antibacterial activity (Radhakrishnan et al., 2018) and good cytocompatibility (Shevtsov et al., 2019). Based on previous studies, a new study was designed to construct AgTa_2_O_5_ nanocomposite films on 316 L stainless steel by magnetron sputtering technology. It was found that, after heating at 400°C, silver ions synergistically enhanced the mechanical properties and antibacterial properties of tantalum coatings (against colibacillus and *Staphylococcus aureus*). Therefore, the technology can promote the preparation of surgical instrument coatings based on nano-composite film (Alias et al., 2020). Owing to their different properties, different ions often induce different changes in the performance of implants, and the combination with calcium or magnesium plasma may be another good choice.

### 3.3 Anodic oxidation technique

In anodic oxidation, tantalum metal is used as the anode; silver, platinum, and other metals as the cathode; and an electrolyte is placed between the two to form a battery path. When a voltage is applied to the electrolyte, an oxidation reaction occurs on the surface of the anode metal, forming a dense tantalum oxide film that effectively inhibits the release of metal ions, enhances corrosion resistance, and has cellular biological activity. These nanoscale tantalum oxide arrays have also been used by some researchers for ion transfer and drug loading (Li et al., 2016). The properties of the oxide layer are mainly adjusted by changing the current power, anodic oxidation time, temperature, applied potential, and chemical composition of electrolytic liquefaction. Anodic oxidation is an available scheme to produce regular nanoscale structures and has been successfully applied to many metal surfaces (Dias-Netipanyj et al., 2020).

The nanoscale porous oxide layer can improve the surface wettability and adsorption capacity of proteins, ions, and cells by broadening the contact area of the material. Some researchers (Liu et al., 2016) co-cultured MG-63 osteoblasts with nano-tantalum implants prepared by anodic oxidation technology. Various osteogenic indexes in the tantalum group were superior to those in the titanium group at each time point. In short, nano-tantalum implants induced more proliferation and differentiation of osteoblasts. With the development of bionic medicine, some researchers combined anodic oxidation and atmospheric plasma spraying technology to develop micro/nano-hierarchically structured tantalum. This kind of layered micro/nanostructure similar to natural bone tissue has great potential in improving cell functions. Moreover, it is clear that the diameter of the nanotube is 15 nm and the length is 800 nm. The layered tantalum can not only reduce the release of metal ions and improve the corrosion resistance of the material by as much as one order of magnitude, but also promote the differentiation of human bone marrow mesenchymal stem cells (hBMSCs) and increase the expression of osteogenic genes by 1.5–2.1 times (Ding et al., 2018). Nano-tantalum prepared by this technique has not been studied *in vivo*, so such studies need to be implemented.

### 3.4 Micro-arc oxidation technology

MAO is an electrochemical technique that generates oxide ceramic film directly on metal surfaces through a high-voltage arc. This effect of high-temperature and high-pressure instant sintering can change the oxide phase on the metal surface and form a more stable crystal phase, which is called the ceramic phase. By controlling the reaction parameters of MAO, such as voltage intensity and treatment time, uniform nanoscale structures can be further constructed on micron-scale surfaces constructed by the implant (Li et al., 2016). Anodic oxidation and MAO are both electrochemical methods. MAO is a surface treatment technology developed on the basis of anodizing. MAO is superior to anodic oxidation in all aspects. The surface layer prepared by this technology has good corrosion resistance and wear resistance, and does not easily detach.

To obtain good surface nano-morphology, a variety of technologies are often combined. High-energy ball milling (HEBM) and pulsed plasma sintering (PPS) combined with MAO could prepare nanocrystal tantalum coating with an oxide layer thickness of 3–4 μm, and the roughness, corrosion resistance, and hydrophilicity of the oxide layer were optimized. MAO nanocrystalline Ta could significantly inhibit *Staphylococcus aureus*, while there was no significant difference between MAO microcrystalline tantalum and nanocrystalline tantalum with regard to *Pseudomonas aeruginosa*, which may be related to the size and shape of the microorganisms. To obtain good antibacterial properties, it is necessary to prepare materials that have wider effects on different strains, such as by introducing Ag and Zn (Sopata et al., 2021). Subsequently, a study combined MAO and DC magnetron sputtering technology to prepare a uniform bionic micro/nano-porous calcium-phosphorus layer containing Zn/ZnO nanoparticles. TACAP-Zn and TACAP-ZnC were classified according to whether additional thin carbon layers were deposited on the nanoparticles. TACAP-ZnC showed better cell adhesion and proliferation because the carbon coating provided a smooth, cell-friendly morphology. Both TACAP-Zn and TACAP-ZnC inhibited the activity of *S. aureus*, but only TACAP-Zn inhibited sessile bacteria. The combination of the two technologies promoted tantalum bone integration and prevented initial bacterial colonization (Fialho et al., 2021). Osteogenesis is coordinated by multiple physiological systems. In addition to antibacterial activities, bone immune regulation also plays a key role in this process. In addition to improving the surface roughness, corrosion resistance, and hydrophilicity, the surface treated with MAO at 300 V promoted the proliferation and differentiation of osteoid Saos-2 cells, and had stronger bone immune regulation (Huang et al., 2018).

### 3.5 Laser cladding technology

Laser cladding technology requires that a layer of cladding material of 1–2 mm be coated on the surface of the implant, and then the cladding layer is melted together with the surface of the implant matrix by a high-energy laser, so as to form an alloy layer with different ion structures on the surface of the implant matrix. The alloy layer can clearly improve the surface properties of the implant. By introducing specific ions into the alloy layer, the layer can have different biological properties. Laser cladding technology makes the bonding between coating and implant substrate closer and effectively solves the problem of ion coating stripping.

A type of gradient nano-porous tantalum scaffold (P-TA-NT) simulating the natural bone structure was also prepared by combining laser cladding and anodic oxidation. In addition to verifying that the gradient nanostructure enhanced the hydrophilicity and protein adsorption capacity, and promoted osteogenic differentiation, animal experiments were also performed. Two weeks after P-TA-NT was implanted into the femur of New Zealand white rabbits, histological examination showed that the P-TA-NT group had improved early bone binding compared with the control group. This showed that nano-porous tantalum scaffolds with a bionic layered structure have considerable application potential (Zhang Z. et al., 2021).

### 3.6 Severe plastic deformation

All of the above methods are “bottom-up” methods. Here, the classic “top-down” methods are presented. One of these is severe plastic deformation (SPD), a technical method to prepare ultra-fine grain (<1 μm) materials by introducing large strain variables in the process of deformation. Compared with the conventional plastic deformation method, the SPD mostly produces larger deformation through strain accumulation, which can effectively refine the metal and obtain sub-micron or even nanometer-scale grains (Zhang et al., 2018). Accumulative roll bonding (ARB), high-pressure torsion (HPT), and equal channel angular pressing (ECAP) are common SPD technologies. Their main advantage is that they can achieve large variables without markedly changing the overall size of the workpiece, among which ECAP is currently the most mature SPD technology. Studies have been performed on composite tantalum nanomaterials prepared by accumulative superpressing and high-pressure torsion methods, which show good mechanical properties and corrosion resistance. However, to the best of our knowledge, few studies have focused on the molecular biological properties of this technology to materials (Raducanu et al., 2011; Yilmazer et al., 2012).

#### 3.6.1 Equal channel angular pressing

ECAP is also one of the classical methods of SPD. The sample is put into an extrusion die composed of two intersecting equal channels, and is then pressed into the channel by a puncher pin pressure at a constant speed; uniform and violent shear deformation is then produced at the corner of the sample. Because the cross-sectional area of the sample before and after extrusion remains unchanged, it can be repeatedly extruded, so that the amount of deformation accumulates and overlaps to obtain greater deformation. Before entering the next ECAP channel, the sample can rotate a certain angle along the central axis of symmetry to produce different paths (Segal et al., 1981). Different ECAP paths, mold angles ψ and φ, friction coefficients, extrusion temperatures, and extrusion speeds have a direct influence on ECAP deformation.

Researchers compared the biological properties of layered walls of different thicknesses (40 and 70 nm, referred to as Ta40 and Ta70, respectively) in gradient nanostructured tantalum prepared by ECAP. They found that the Ta40 surface with thinner nanolayers had the strongest effects of promoting cell proliferation and differentiation, while greater protein adsorption and β1 integrin expression were also observed. Atomic force microscopy showed that Ta40 had the strongest adhesion to osteoblasts, due to an increased contact area between cells and the Ta40 surface. The great potential of gradient nanotantalum structures was again confirmed (An et al., 2019). In addition to promoting osteogenic differentiation, vascular compatibility was also explored. It was shown that an individual BCC microstructure with an average grain size of about 220 nm was successfully fabricated by the ECAP technique until eight passes, which showed excellent cytocompatibility with the L929 cell line. It was also illustrated that the hemolysis rate and the number and state of adherent platelets decreased with decreasing grain size. The ECAPed Ta accelerates hemocompatibility, and should play an important role in the fields of surgery and transplantation (Nie et al., 2014).

The plastic deformation technique is an efficient method for grain refinement. However, the grain size does not change with increasing strain when the grain size is refined to a certain size under specific deformation conditions and deformation modes. In recent years, Lu et al. successively developed several new deformation methods to improve the limitations of the above techniques.

#### 3.6.2 Surface mechanical attrition treatment

SMAT was developed by Lu et al., after which the concept of surface nanocrystallization was also proposed. This involves the original coarse grain on the sample surface being refined to nanometer scale by the plastic deformation method, while the core retains its original structure (Li et al., 2008). This method can achieve a faster rate of deformation. The equipment mainly includes a vibration generator to make the spherical projectile resonate and hit the surface of the sample constantly at a certain speed and different angles so that plastic deformation occurs. The sample surface would normally have the greatest strain rate. As a result, the grain size can be refined to nanometer scale. With increasing depth, the strain variable and strain rate gradually decrease, and the grain size gradually increases until the original structure of the core. SMAT is considered as a SPD method, which can effectively improve the surface-related mechanical properties of traditional metallic biomaterials (Lu and Lu, 2004). Nanolamellar structures with a stable and small-angle grain boundary can be prepared by this method, which overcomes the limit of grain refinement by plastic deformation technology. On this basis, surface mechanical grinding technology (SMGT) developed by Lu et al. can realize grinding on the basis of pressing. Generally speaking, the sample deformation layer processed by SMGT technology is significantly deeper than that of SMAT, which can meet some industrial requirements. Stable gradient nanostructures have been successfully prepared on the surface of a series of metals such as copper and nickel by this technique (Hou et al., 2021).

The physical, electrochemical, tribological, and biological properties of a new low-modulus β Ti-Nb-Ta-O alloy prepared by SMAT have been studied. This alloy has two different microstructures, namely, single-phase β treatment and dual-phase β+α aging. After SMAT, the corrosion rate, the friction coefficient, and the wear volume loss were found to decrease, while the surface toughness increased. However, SMAT did not affect the adhesion and growth of osteoblasts *in vitro*, which were still comparable to those with pure titanium. These results highlight the importance of initial microstructure in determining the properties of alloys (Acharya et al., 2020). To further clarify the capabilities of SMAT, more and improved molecular studies of osteogenesis need to be performed.

#### 3.6.3 Sliding friction treatment

Zhang et al. developed a surface nanocrystallization technique called sliding friction treatment, which utilizes a ball-on-disc contact structure and is characterized by a large sliding amplitude and increased controllable contact conditions to expand the sample size. It is also a low-cost, simple, and efficient technique for performing severe plastic deformation. A specially designed device is used for the ball-on-disc contact configuration, as shown in Figure 1. The WC-CO ball with a diameter of 10 mm is static. The sample is firmly installed on the workbench. Under the action of a 100–500 N pre-managed force, the WC-CO ball and specimen are pressed together. The table is driven by two motors and moves independently along the X- and Y-axes. First, the sample slides along the X-axis with a velocity of V1 relative to the WC-Co ball with an amplitude of d1. Then, the table with the sample moves along the Y-axis with an amplitude of d2. The sliding process continues until the area on the surface of the sample is sliding treated (Zhang et al., 2014).

Soon after, the team (Huo et al., 2017) employed SFT technology to prepare a new nanosurface layer with average grain size of ≤20 nm on pure tantalum, and tested its biological properties. They maintained that the novel nanocrystalline tantalum had excellent corrosion resistance and yield strength, and good surface energy and hydrophilicity, which also contribute to protein adsorption, showing advantages in cell adhesion, proliferation, and bone formation. Through detection of the expression of different osteogenesis-related genes, it was suggested that the nanotantalum surface promoted osteogenic differentiation by promoting an early maturation phenotype. Nanosurface tantalum made by SFT is expected to be applied in load-bearing bone implants.

## 4 Surface functionalization involving bioactive ingredients

**FIGURE 2:**
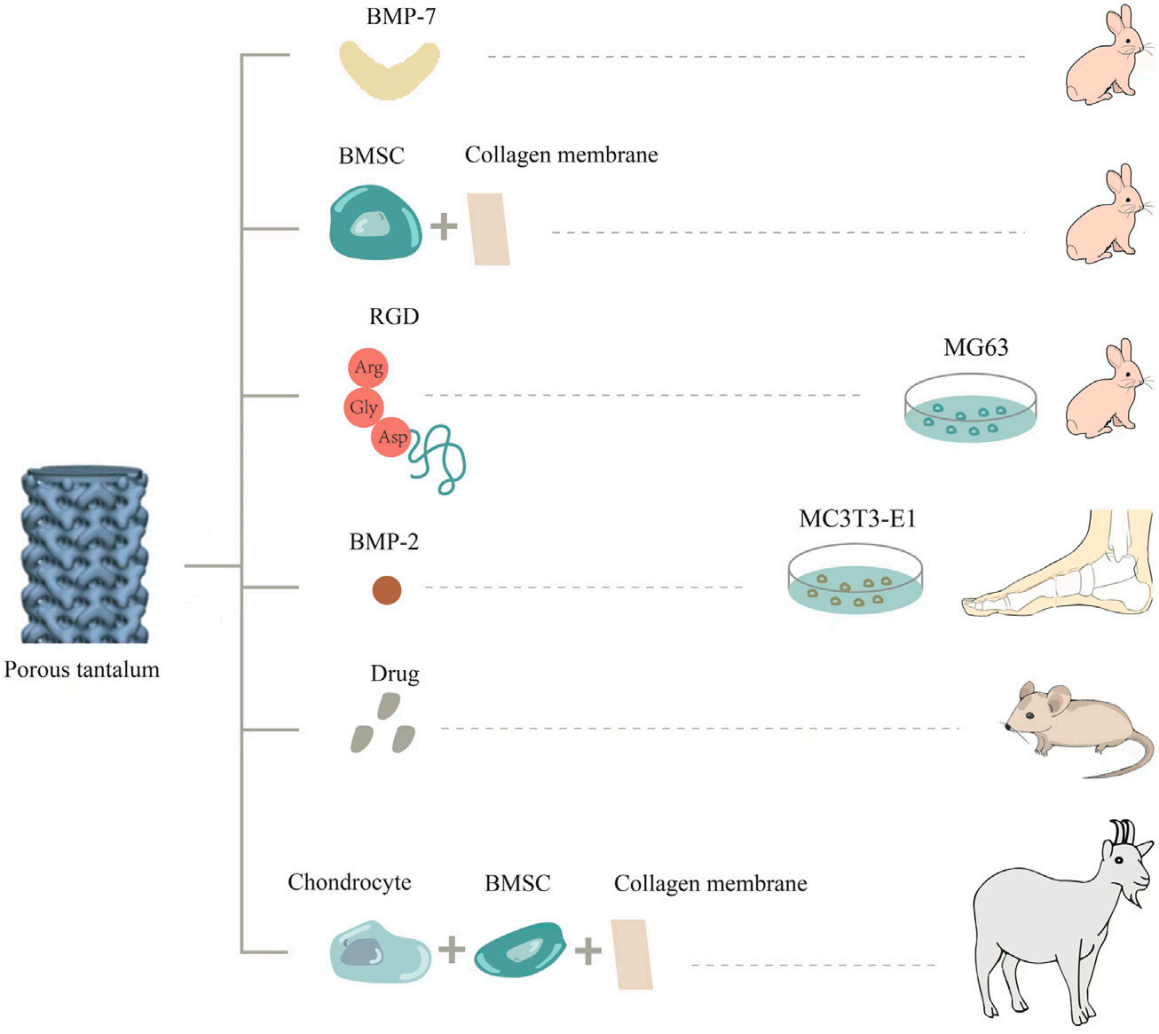
Surface functionalization involving bioactive ingredients of porous tantalum.

Ideal functionalization modification involving bioactive ingredients refers to the uniform attachment of natural or artificial bioactive ingredients to the surface of a metal, and the stable release of natural or artificial bioactive ingredients in the body in a localized and controlled way (Figure 2). The minimal effective dose directly acts on the adjacent tissue cells to avoid toxic and side effects, thus realizing the functionalization of metal materials. For example, bioactive ingredients such as cells, extracellular matrix proteins, growth factors, and drugs can be loaded onto the surface of porous materials to make them functional biomaterials (Souza et al., 2019). These biofunctional coatings are applied to improve bone integration as well as the integration of soft tissue around implants, thereby decreasing the risk of peri-implant inflammation resulting from biofilms (García-Gareta et al., 2017). In particular, porous tantalum is more conducive to loading due to its special scaffold structure to achieve specific biological functions.

In a related study, porous tantalum and Bio-Gide collagen membrane were co-cultured with BMSCs *in vitro*, and then implanted into the femoral head defect area in a rabbit model. Twelve weeks after surgery, thick hyaloid cartilage was detected at the top of the tantalum near the defect edge. In addition, scanning electron microscopy (SEM) revealed that the structure promoted the secretion and penetration of nutrients and metabolites, as well as larger bone growth (Liu et al., 2019). Similarly, researchers also constructed chondrocyte/collagen membrane-BMSCs/porous tantalum (pTa) composite material, and implanted it into goats for 16 weeks. It was found that the composite material had good repair effects and good biocompatibility. It can promote the adhesion and growth of BMSCs and chondrocytes, and the high expression of ALP, OSX, OCN, COL-1, OSN, and RUNX2, as well as of the chondrogenic genes COL-II, Agg, and SOX9g. This provides a new, efficient, simple, and safe option for treating large bone injuries in load-bearing areas (Wei et al., 2019).

Bone morphogenetic protein-2 (BMP-2) is the most significant growth factor that promotes osteoblast differentiation and induces bone formation (Tang W. et al., 2016). Yu et al. prepared graded porous tantalum coated with bioglass membrane loaded with BMP-2 by a sol-gel process and found that porous tantalum functionalized by BMP2 could enhance the osteogenic differentiation of MC3T3-E1 cells (Yu et al., 2017). On the basis of cell experiments, some researchers carried out studies *in vivo*. As a member of the bone morphogenetic protein family, BMP-7 has a strong ability to promote ectopic osteogenesis and can constitute ectopic new bone, which plays an important role in bone development, bone defect repair, and cartilage differentiation (Tóth et al., 2021). Porous tantalum loaded with BMP-7 was implanted into a rabbit model of femoral condylar cartilage defect. At the fourth, eighth, and 16th weeks after surgery, porous tantalum loaded with BMP-7 formed more cartilage and bone tissue, and the volume fraction of new bone, the quantity and quality of bone trabecula, and the maximum release force of bone were higher (Wang et al., 2018). In addition, Kreulen et al. implanted porous tantalum supplemented with bone morphogenetic protein 2 (BMP-2) into the human ankle to promote bone fusion. At an early stage of 4–6 weeks postoperatively, complete bone fusion occurred at the implant-bone interface, and no failure was observed (Kreulen et al., 2016).

Arg-Gly-Asp polypeptides (RGD) are present in extracellular matrix proteins such as fibronectin, osteopontin, and sialoprotein, which can be used as recognition sites for the binding of integrin receptors on cell membranes to extracellular ligands. In some studies, porous tantalum was modified with these polypeptides and co-cultured with osteoblasts. It was found that the expression of osteoblast-related proteins OC and FN and cytoskeletal protein F-actin was higher than that in the unmodified group. Scanning electron microscopy also showed that the cells adhered and spread well on the porous tantalum modified by RGD, which promoted bone integration (Gan et al., 2018). In addition, the research team implanted porous tantalum scaffolds treated with RGD into a rabbit segmental bone defect model. X-ray and histological observations were performed to assess bone repair at three different postoperative time points. The results indicated that bone formation was increased at the interface and internal pores of treated porous tantalum scaffolds (Wang et al., 2017). Recently, the team also elucidated that RGD polypeptide modification of porous tantalum can upregulate the expression of type col-1 and integrin β1 in cells, and then activate the focal adhesion kinase signaling pathway to promote cell adhesion.

The treatment of bone and joint *tuberculosis* remains a clinical challenge. Porous tantalum surface-loaded drugs satisfy the requirements of good bone biomechanics, cytocompatibility, and antibacterial function. Some studies have loaded porous tantalum with the anti-tuberculosis drugs isoniazid and rifampin, and treated the 3D-printed porous tantalum surface with polydopamine to increase adhesion. *In vitro* and *in vivo* studies have shown that the drug is released slowly, increasing the duration of antibacterial action. This composite biological scaffold inhibits the growth of *Staphylococcus aureus* and has good biocompatibility. This scaffold can also realize local long-term controlled drug release and bone regeneration at the same time, which is a promising approach for treating osteoarticular *tuberculosis* (Hua et al., 2022). The loading of many drugs on porous tantalum has been studied, such as vancomycin (Sautet et al., 2019), alendronate (Garbuz et al., 2008), and PHA containing gentamicin (Rodriguez-Contreras et al., 2019), with good results being obtained.

## 5 Bionic surface coating

To enhance the biological activity of the implant material, the metal surface is often covered with a layer of bioactive material with good bone integration ability, which mainly includes calcium phosphate and inorganic metal elements. Based on the principle of heterogeneous nucleation, the implant is immersed in a supersaturated solution of calcium phosphate, which nucleates on its surface and forms a coating. Calcium phosphate materials have physical and chemical properties similar to those of natural bones in the body. They are also thought to possess excellent bone conductivity and improve inward bone growth. Researchers have applied calcium phosphate coatings to substrates through a variety of methods, such as plasma spraying (Lu R.-J. et al., 2019), magnetron sputtering technology (Cao et al., 2021), electrochemical deposition (Azem et al., 2016), ion immersion injection (Lu et al., 2016), and the sol-gel method (Maho et al., 2013).

Antonio et al. recently investigated the surface morphology and chemical composition of hydroxyapatite coatings deposited on tantalum by plasma electrolytic oxidation (PEO), and found that the surface properties were strongly influenced by treatment parameters (Antonio et al., 2019). Colorimetric quantification of MTT metabolic oxidation and alkaline phosphatase activity confirmed that hydroxyapatite-modified tantalum increased the surface bioactivity compared with raw tantalum, resulting in enhanced alkaline phosphatase activity. In addition, X-ray diffraction confirmed that samples treated for more than 180 s at 500 V contained up to 80% hydroxyapatite coating, resulting in improved surface bioactivity. Through *in vivo* animal experiments, Barrere’s team (Barrere et al., 2003a; Barrere et al., 2003b) implanted cylindrical porous tantalum implants with a bionic coating of calcium phosphate into the back muscle and femur of goats, and observed good osteogenesis effects. The contact area between the implant and bone tissue was larger than that of the uncoated implant.

In addition, in some studies, metal elements such as strontium (Cheng et al., 2021), copper (Wu et al., 2021), and boride (Wang P. et al., 2020) were mixed into the tantalum surface, and all of the BMSCs implanted on the tantalum surface showed good proliferation and adhesion. Tantalum surfaces doped with the above metal elements can promote osteogenesis and angiogenesis, and inhibit inflammation.

Owing to its excellent biological properties, tantalum is often combined with other elements to form coatings with improved performance by many fabrication methods, which cover the surface of various materials and achieve good results (Table 1).

**TABLE 1:** Tantalum is combined with other factors to form a coating.

Fabrication method	Base material	Components	Key results	Cell	References
Chemical vapor deposition	Porous titanium	Tantalum coating	Enhanced cell proliferation and adhesion	MG-63 cells	Liu et al. (2022)
Themal synthesis + magnetron sputtering	Magnesium matrix	Tantalum + FHA	Enhanced cell viability; Proliferation; Osteogenesis; Differentiation	MC3T3-E1 cells	Cao et al. (2021)
Vacuum evaporation	PEEK	Tantalum coating	Enhanced cell proliferation And adhesion; Osteogenesis; Differentiation	BMSCs, HGE cells	Pang et al. (2021)
Plasma spraying	PEEK	Tantalum coating	Active the BMP2 osteogenic pathway	BMSCs	Lu et al. (2018)
Plasma spraying	Titanium alloy	Tantalum coating	Enhanced cell proliferation and adhesion; Osteogenesis Differentiation	MC3T3-E1 cells	Wang et al. (2020a)
Plasma spraying	Titanium matrix	Tantalum + HA	Enhanced mechanical properties and osteogenic activity	BMSCs	Lu et al. (2019b)
S-PIII	PLA	Tantalum	Enhanced osseointegration		Park et al. (2019b)
Magnetron sputtering	PLA	Tantalum	Enhanced cell proliferation and adhesion; Osteogenesis; Differentiation	MC3T3-E1 cells	Hwang et al. (2020)

## 6 Discussion

Tantalum as a bone replacement material has shown surprising biological properties. Porous tantalum, which has been applied in many clinical fields and achieved the effect of promoting osteogensis (Li Z. et al., 2020). How to further improve the biological properties of tantalum, promote the integration efficiency of the tantalum-cell-bone interface, and increase the value-added utilization of tantalum have become hot research topics. In this review, we have discussed the physicochemical properties and biological performance, and summarized the classical surface modification methods of tantalum for the first time. These classical tantalum surface modification methods can be mainly divided into “top-down” and “bottom-up” approaches. Although tantalum surface modification technology has been around for a long time and is constantly being improved, most related studies remain at the level of *in vitro* cell experiments, while many obstacles remain to be overcome.

First, the difference in mechanical strength between coating and metal leads to poor stability and easy stripping of the coating. The microporous structure on the surface of porous tantalum implant material is conducive to the loading of various cytokines or drugs. How to maintain the biological activity and stability of the coating and control the dynamics of its slow release are difficult issues. Second, owing to the complex structure of the human body, the morphology of implant materials also varies. How to conduct uniform nano-processing on the surface of implant materials with complex morphology is also a challenge. EDM is a classical surface treatment method, that can improve the corrosion resistance and fatigue resistance of the implant material, which is very important for the long-term stability of metal implants in the body. However, the application of EDM to tantalum surfaces remains to be explored, which also expands the direction of our future research.

**FIGURE 3:**
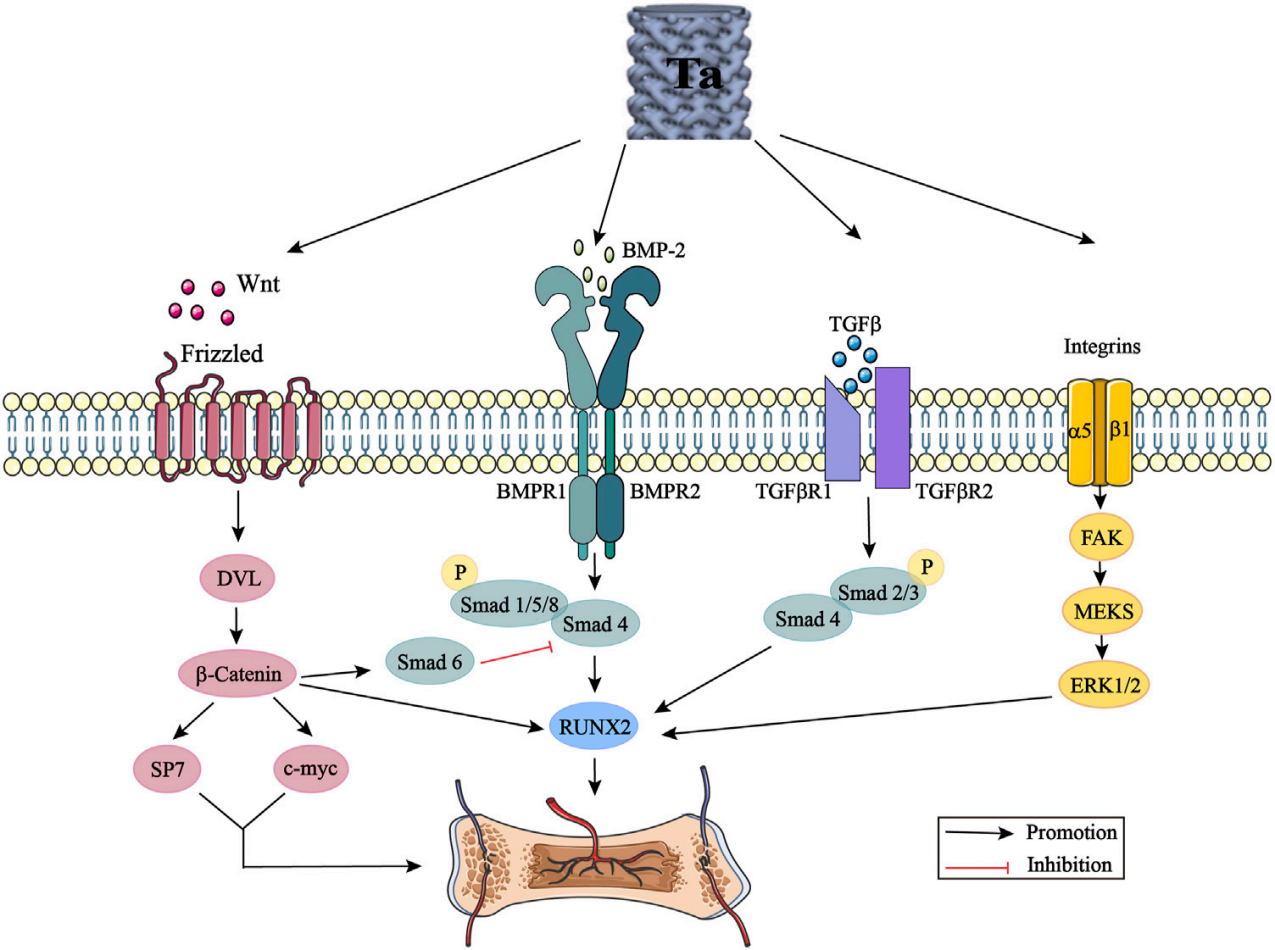
Tantalum activates the Wnt/β-catenin signaling pathway, BMP signaling pathway, TGF-β signaling pathway and integrin signaling pathway by promoting the expression of Wnt, BMP-2, TGF-β and integrins. DVL: disheveled; BMP: bone morphogenic proteins; Smad: small mother against decapentaplegic; Runx2: runt-related transcription factor 2; TGF-β: transforming growth factor-beta; FAK: focal adhesion kinase; ERK: extracellular signal regulated kinase.

At present, although the clinical application of tantalum has achieved good short-term efficacy, some studies have also clarified the potential partial osteogenic mechanism of tantalum through some classical signaling pathways, including the Wnt/β-catenin signaling pathway, BMP signaling pathway, TGF-β signaling pathway, and integrin signaling pathway (Figure 3). However, few biological studies related to tantalum and involving in-depth exploration of its mechanism of action have been performed, and there are complex cross-talk effects among these signaling pathways. More comprehensive, detailed, and in-depth mechanistic exploration, *in vivo* study of the nanocrystalline structure of the tantalum surface, and long-term comparison of clinical efficacy still need to be carried out. With the development of proteomics and genomics, we can also broaden our range of approaches for exploring the mechanism of tantalum osteogenesis. In future research, we aim to further study the mechanism of osteogenesis. Finally, the widespread application of tantalum is limited by its high cost and complex production process. Most of the materials prepared by violent plastic deformation technology are small in size, with the preparation of large-size materials remaining difficult. There is a need to invest in substantial research to explore the size limit and to prepare surface nano-tantalum with the optimal size and thickness. The development of tantalum implant materials with simple preparation methods, low cost and excellent performance, and the further exploration of its osteogenesis mechanism are the hot topics of future research. Comprehensive research is required, involving the cooperation between materials science, biomedical and manufacturing researchers.

## 7 Conclusion

Various tantalum surface modification methods are available, which provide a variety of directions for research on tantalum interfaces. This paper summarizes the nano-modification, surface functionalization involving bioactive ingredients, and bionic coating of tantalum surfaces, and expounds the characteristics and value-added effects of the various methods available to further broaden the range of ideas for tantalum value-added utilization. It is believed that tantalum will be widely used in clinical medicine with the development of production technology and continuous improvement of experimental evidence supporting its use.
